# Integrating father involvement into early childhood initiatives delivered at scale: key considerations

**DOI:** 10.3389/fpubh.2023.1193974

**Published:** 2023-12-19

**Authors:** Sapna Nair, Harshula Sinha, Penny Holding

**Affiliations:** ^1^Institute for Financial Management and Research, Krea University, Chennai, India; ^2^Datta Meghe Institute of Higher Education and Research, Wardha, Maharashtra, India; ^3^Identitea, Nairobi, Kenya

**Keywords:** childcare, early child development, father, father involvement, ECD intervention childcare, ECD intervention

## Abstract

Early child development (ECD) interventions, usually centered around the mother, exclude fathers from contributing to childcare. Research shows that a father’s involvement in childcare can have a positive impact on the child’s growth. In this light, a trial was conducted in rural India wherein a new program component aimed at enhancing fathers’ engagement in early child was incorporated into an existing childcare intervention. The paper highlights the learnings drawn from the trial to present pathways to change, that is, strategies needed to embed father involvement as a component within the intervention ecosystem.

## Current advances and future directions in the field

The first trial in rural India to enhance fathers’ engagement in early childcare was carried out by The Institute for Financial Management and Research-Leveraging Evidence for Access and Development (1, 2). The intervention added outreach to parents to the existing Integrated Child Development Services (ICDS). ICDS is the nationwide flagship program supporting children under the age of 6. It is a classic female-centric welfare program where the mother is the primary recipient, and the program is implemented by an exclusively female cadre called the Anganwadi worker (AWW). Since its inception in 1975, the program has undergone various changes in design but is yet to carve out a space for fathers to engage in their child’s development.

Without an active component to involve fathers, the program loses out on the opportunity to nurture the idea of family as a unit of care, and excludes men from meaningfully contributing to their child’s development. Research shows that fathers’ active involvement in childcare has a positive impact on the child’s cognitive, physical, social and emotional development (3). On the other hand, child development is adversely affected by fathers’ emotional unavailability, anger, and physical and verbal hostility, especially physical aggression toward the mother (4).

Interventions engaging men and boys can usher positive changes in gender role attitudes, gender-based violence, quality of relationship, and equitable division of household responsibilities (5, 6).

However, evidence of impact alone cannot guide the changes in behavior required to shift a system of practice. In addition to a shared understanding of whether it works or not, awareness of effective processes and of the pathways to change are equally important to the success of any intervention. In addressing sexual and reproductive health and rights intentional and explicit promotion of gender equality and gender-transformative programming is required (7). According to Ruane-McAteer et al. (7), interventions require a closer examination of the aims, the theory of change, and whether there is explicit attention to issues of male privilege, power and position in relation to women. Simply put, it is not just about what to do, but also how to do it and how to remain effective over time.

The major challenge of evidence of impact not directly translating to improved implementation is a universal problem. While it is well established that a father’s positive engagement in physical-emotional-nurturing care can contribute to a child’s holistic development (8–10), this knowledge has not consistently driven implementation design. Child development interventions continue to exclude fathers and do not address the gendered difference in caregiving and parenting practices. To change this dominant narrative, more intentional activities need to be implemented, carefully monitored, and evaluated to provide the shared learning that drives transformational change.

In view of this body of evidence, the LEAD initiative centered on engaging both parents in childcare through information technology, group activities, and incentives delivered through the network of AWWs (1). The complexity of community-based development projects, with multi-stakeholders and interest groups, demands constant reflection and adaptability to circumstances to make meaningful change. In this paper, we take forward our exploration of the evidence to landscape father involvement in the childcare paradigm in India. We reflect on the experience of the LEAD initiative and endeavor to understand the efforts of other practitioners as we critically evaluate potential next steps in scaling the integration of fathers into the ICDS, and other similar programs.

## Authors’ perspectives

### Reflections on the current system of practice

We use the Measurement for Change (M4C) framework (11–13) to guide our retrospective on outcomes and process. The M4C framework draws attention to aspects of design and information utilization that have guided effective delivery. These have been labeled as *Dynamic* (responding to the feedback loop), *Informative* (the triangulation of sources), *Inclusive* (the collaborative-co-creative process), *Interactions* (awareness of intended and unintended consequences), and *People-Centered* (going beyond the average to understand and address variability in outcome). Within this framework, the review involves exploration of the assumptions of our initial design vis-à-vis requirements for scaling of the model. This process is guided by learnings from existing studies and reflections gleaned from discussions with intervention practitioners.

#### Informative

In our reflective process, we sought experiences and insights from practices promoting father involvement in India. We did not find any published literature, but were able, through our networks, to gather the evidence of practitioners who have integrated fathers into early child development (ECD) initiatives. One source was Ashoka University, who had undertaken the Cognitive Science Research Initiative to explore the relationship between mother–child interaction and cognitive development in at-risk infants (14). The attendance of a few fathers in the study sessions revealed positive interactions between fathers and their infants and highlighted differences between maternal and paternal interactions (Madhavi Maganti personal communication). Other interventions such as the Daddy Cool campaign, a CSR initiative implemented in the urban slums of Lucknow, used radio and social media targeting to improve the engagement of fathers in ECD activities (15). Within the ICDS too, there have been local level improvisations to include fathers in household chores and child nutrition, through public meetings and home visits (16). Whether part of a more experimental design or community-based outreach, these initiatives were perceived to have made a discernible change in the interactions between families and children. However, information was drawn largely from anecdotal evidence. The lack of more rigorous or systemic data amounts to a missed opportunity to gather evidence that could advocate for specific strategies and changes to policy and practice. These missed opportunities are all the more important in the light of the evidence of the multilevel impact that fathers can have on child development outcomes observed in the IFMR-LEAD intervention (2).

#### Inclusive

LEAD intended to provide a template for scaling father involvement strategies through the ICDS platform. To create this template, we documented the responses to the pilot across the network of partners and actors in the system. It was important to gather evidence from multiple perspectives to address the fit to the Indian context of engaging fathers in interventions on nurturing care. The questions asked focused on understanding the current state of fathers’ engagement in rural areas; areas of childcare where the fathers’ role can be leveraged; and effective strategies to influence cultural norms surrounding fathers’ role in childcare. Strategies to engage fathers and other family members in the overall improvement in ECD outcomes were then developed in collaboration with participants and incorporated into the template.

#### Interactions

To examine influences upon the scaling of the LEAD initiative and the framing of policy to support this, we mapped the reflections from invested communities, and the interactions between the network of participants (program staff, IFMR-LEAD, funders, health care workers, rural communities and, most important of all, the ICDS program). Evidence from the literature from India indicates that any such intervention is likely to clash with the patriarchal structure of society as patriarchal models of masculinity predominantly shape fatherhood and continue to dominate the structure of the working environment and the design of support services for nurturing care (17). Hence, father’s involvement in childcare is not affected by situational factors like the mother’s employment status (18), but is rather determined by gender role expectations, perception of the peer group, and the fathering that they received (19). Even in urban India where desire and demand for greater involvement by fathers is rising, their role as providers take precedence, with working hours keeping fathers from participating in shared activities with their children (8). As a result, childrearing remains primarily a mother’s responsibility, while fathers’ roles remain limited by their social and professional environment. To address the root of the issue, societal pressures, more positive and inclusive messages to provide the space for behavior change were framed, which present interactive parenting and the family as an effective secure unit for providing childcare.

#### People-centered

Through our reflection, we have found navigating competing priorities within and between actors in the implementation system to be a major challenge. The success of a specific intervention is strongly influenced by the importance accorded to the proposed change against competing challenges. Given that the ICDS program is already considered successful in tackling undernutrition and is delivered by a complex mechanism, there was an obvious reluctance from the practice community perspective to introduce additional changes to the existing intervention. This resistance to change came from competing choices in terms of value systems and the goals of research. The question then becomes how important is the change in the overall scope of changing lives and improving interventions, and how visible the improvements are to those invested in policy design. Clear and memorable data that informs the wider picture and details practical experiences can play a crucial role in the advocacy process as well as in guiding policy makers and the practice community. Most importantly, the pilot underscored the need for adopting an attitude of being continually adaptive to the interests of the community of practice and the community of intervention.

#### Dynamic

So far, the ICDS program appears to have been successful in reducing undernutrition and growth failure among women and young children, especially in the rural areas of India (20). Yet gaps are emerging, even in the relative success of the nutritional components (21). Moreover, not only is there scant evidence to prove the efficacy of the impact upon more holistic ECD outcomes (22), but there have also been no evaluations of the various programmatic components. Questions remain unanswered on how service efficiency can be achieved and how to ensure durable change across all components of child development. In light of the changing landscape of ECD requirements, the program needs to be continuously responsive to emergent challenges, as well to new evidence that can improve program design and enhance effectiveness.

## Driving sustainability

In this section, we turn to the LADDERS elements, *Leadership, Alignment, Data, Demonstration, Evaluation, Replication,* and *Sustainability* (23) to reflect on the activities required to build a sustainable process for integrating father involvement strategies into the ICDS public programs.

When we reflect upon the *Leadership* required to drive the integration process the key gatekeepers are the senior officials in the Government departments through which the centrally envisioned ICDS programs are implemented. System readiness has been observed to target fathers directly in a nutrition initiative, Poshan Pakhwada, with the adoption of the slogan of “Men for Nutrition” (16). The attention on engaging fathers for improving only the nutritional indicators misses the opportunity for a sustained engagement strategy across holistic nurturing care. Nevertheless, it indicates a gradual shift toward an awareness of the importance of engaging the father. To continue this process, information must particularly reach the high-level decision-makers in the Women and Child/Social Welfare Departments at the state level.

Targeting the leadership alone to support the process of change will, however, be insufficient to create lasting change. To overcome the challenge of competing goals *Alignment* across the system requires adaptive processes throughout, with the entire system collaborating around common goals and objectives. Including fathers in outreach and information strategies seems like a small component in the entire umbrella of ICDS activities, yet integration requires multiple additional activities for it to work successfully. Each activity needs to be accepted and integrated into the existing implementation process, with training and supervision seamlessly linked at multiple levels. An inclusive process of monitoring, evaluation, and learning will play a central role in this integration.

Another influence on system transformation is managing the variations found across contexts. Many of the maternal child health projects in India follow a hub and spoke model, centrally designed and monitored but with the state in charge of the implementation. Successful *Replication* across different sites, however, depends on the ability to adapt to, and through, local contextual knowledge, while in turn providing the model for innovations adopted centrally. The LEAD project, for example, was run in the state of Tamil Nadu, which has historically initiated many innovations in social welfare, including the ICDS supplementary nutrition project later rolled out at the national level. In response to the evidence of the LEAD project, other states, including New Delhi and Haryana have shown a willingness to adopt the integration of father involvement strategies into the ICDS. The transfer of knowledge to guide design, therefore, needs to use information that is accessible, drawn from multiple sources, and which flows in multiple directions.

*Demonstration* speaks to the need to learn through doing, and the ability to build in changes in the process. Incentivization is an example of a strategy that has been used successfully to promote the immunization of children in resource-poor settings. The addition of monetary incentives has enhanced immunization rates (24), while a regular supply of immunization services (25), and the use of SMS reminders (26, 27) have also improved delivery processes. Upgrading and extending the information system through the use of tablets, audiovisual communication and incentives can induce interest among the service providers and the target communities. Decisions on what will support delivery and what incentive will be most valued must directly include those who are targeted by the strategy, as monetary incentivization may not be sustainable, and should not be assumed to be universally valued. This suggests that indicators of program success need to broaden in range and scope, and careful advocacy is needed to use this knowledge to engage partners in the process.

To address *Sustainability,* additional components should be both acceptable and empowering to the last-mile worker and targeted families. With centralized design there is limited scope for drastic design change, yet the reality is that the project relies mainly on the last-mile worker, and success depends heavily on incremental change at that level to be sustainable. The key is for any additional activities to demonstrably support overall implementation without placing additional hardship on the last mile, the Anganwadi workers, who are hugely burdened and underpaid. A key ingredient of success is keeping staff motivated to continue to address challenging activities, such as mothers’ gate-keeping behavior, alcoholism among fathers, and rapidly changing phone numbers. The efforts made need to build an increasing professionalism. Progress must be monitored and documented, and learning loops developed within each geographic cluster or state. The feedback loop should circle back to the Government to enable necessary changes within the intervention ecosystem.

Monitoring and Evaluation (M&E) of the ICDS is currently focused on the impact of nutritional supplementation, which is comparatively easy to evaluate using low-cost nutritional outcome measures among children. Information on the effective integration of additional components will inevitably make the process of evaluation more complex, requiring a focus on indicators of behavior change. Moreover, evidence derived from practice is more difficult to capture in the existing top-down monitoring and evaluation system. *Data and Evaluation* are, therefore, the most challenging elements of system change and adaptation to context, but vital to drive effective decision-making.

*Data and Evaluation* is not just about the “what” and “how” of collecting information, but also about using it (28). Information systems need a significant redesign to be responsive to questions that arise as system transformation proceeds. Expanding the data system from M&E to MEL or MERL (monitoring, evaluation, research, and learning) requires these systems to be integrated into the implementation process and tied to an effective communication strategy.

## Conclusion

Childcare has long been considered a woman’s role, and this “Culture of Maternalization” has also been responsible for keeping fathers out of child development interventions. These reflections are a call to action to not only leverage fathers’ engagement in childcare interventions but also to implement with consideration for all the moving parts of a new strategy and the adoption of a constant reworking of that strategy.

Our reflections highlight the dynamic nature of the implementation of a new strategy within an existing intervention ecosystem. The inclusion of behavioral change components at the community level is vital, but it must also be recognized that outcomes may be slow to achieve and difficult to perceive or measure. Therefore, tracking and monitoring too must be feasible enough to occur over an extended period of time. To make this process sustainable, we need to strengthen the networks and collaborations between Government agencies, existing ICDS staff, and external implementation and research partners. Technical assistance will be equally vital for training of staff, re-development of apps and modules to suit the state contextually, as well as for gathering and sharing the learnings from the project.

To ensure replicability, the strategies adopted need to be highly adaptive and sensitive to contextual factors. Across contexts, low attendance by fathers and a strong influence of societal gender norms will need to be intentionally addressed (29). Successful replication will also require micro-strategies that can adapt to change. The multiple components of ECD should be introduced into programmatic evaluation, while also moving beyond external evaluation toward data utilization that builds shared accountability and responsibility within the implementation system

## Data availability statement

The original contributions presented in the study are included in the article/supplementary material, further inquiries can be directed to the corresponding author.

## Ethics statement

The studies involving human participants were reviewed and approved by IFMR Human Subjects Committee (dated September 26, 2017). Written informed consent was provided by all participants, the parents in the study. The studies were conducted in accordance with the local legislation and institutional requirements. The participants provided their written informed consent to participate in this study.

## Author contributions

All authors listed have made a substantial, direct, and intellectual contribution to the work and approved it for publication.
